# Concurrent high-grade appendiceal mucinous neoplasm and adenocarcinoma: a unique case report and literature review

**DOI:** 10.1093/jscr/rjaf666

**Published:** 2025-08-29

**Authors:** Mohammed N AlAli, Jawad S Alnajjar, Mohamed S Essa, Arwa F Alrasheed, Ruba M Alzuhairi, Nouf A Alromaih, Sadiq M Amer, Mohammed Sbaih

**Affiliations:** Department of Surgery, Prince Mohammed bin Abdulaziz Hospital, Ministry of Health, Riyadh, Saudi Arabia; College of Medicine, King Faisal University, Alahsa, Saudi Arabia; Department of Surgery, Prince Mohammed bin Abdulaziz Hospital, Ministry of Health, Riyadh, Saudi Arabia; General Surgery Department, Faculty of Medicine, Benha University, Benha Egypt; Department of Surgery, Prince Mohammed bin Abdulaziz Hospital, Ministry of Health, Riyadh, Saudi Arabia; Department of Surgery, Prince Mohammed bin Abdulaziz Hospital, Ministry of Health, Riyadh, Saudi Arabia; Department of Surgery, Prince Mohammed bin Abdulaziz Hospital, Ministry of Health, Riyadh, Saudi Arabia; Department of Pathology, Prince Mohammed bin Abdulaziz Hospital, Ministry of Health, Riyadh, Saudi Arabia; Department of Surgery, Prince Mohammed bin Abdulaziz Hospital, Ministry of Health, Riyadh, Saudi Arabia

**Keywords:** appendiceal neoplasms, adenocarcinoma, appendicitis, gastrointestinal neoplasms, surgical oncology

## Abstract

Appendiceal neoplasms (ANs) are rare and often mimic acute appendicitis, complicating timely diagnosis. We report a unique case of a 57-year-old male presenting with right lower quadrant pain, found on imaging to have a ruptured appendiceal mucocele. He underwent open right hemicolectomy, and histopathology revealed concurrent moderately differentiated mucinous adenocarcinoma arising within a high-grade appendiceal mucinous neoplasm—the first such case documented in the literature. This case underscores the need for early detection, proper surgery, and long-term follow-up in managing rare concurrent appendiceal tumors.

## Introduction

Appendiceal neoplasms (ANs) are rare clinical entities with variable malignant potential [1]. Although the occurrence of a single appendiceal tumor is uncommon, several studies have reported cases of simultaneous ANs in the same patient [2, 3]. However, the incidence of concurrent ANs is unclear due to their rarity. We report the first known case of high-grade appendiceal mucinous neoplasm (HAMN) and adenocarcinoma occurring together, highlighting diagnostic challenges and important clinical implications.

## Case presentation

A 57-year-old male with no known medical conditions and a past surgical history of hemorrhoidectomy presented to the emergency department with a 10-day history of mild, diffuse abdominal pain, which had become more intense and localized to the right lower quadrant (RLQ). He denied any other symptoms. He had previously been diagnosed with a perforated appendiceal mucocele outside the country and was treated conservatively after declining surgical intervention.

On examination, the patient was conscious, alert, and oriented, afebrile, with stable vital signs. Abdominal examination revealed fullness in the RLQ with mild tenderness, but no signs of diffuse peritonitis. Laboratory investigations were within normal ranges.

**Figure 1:**
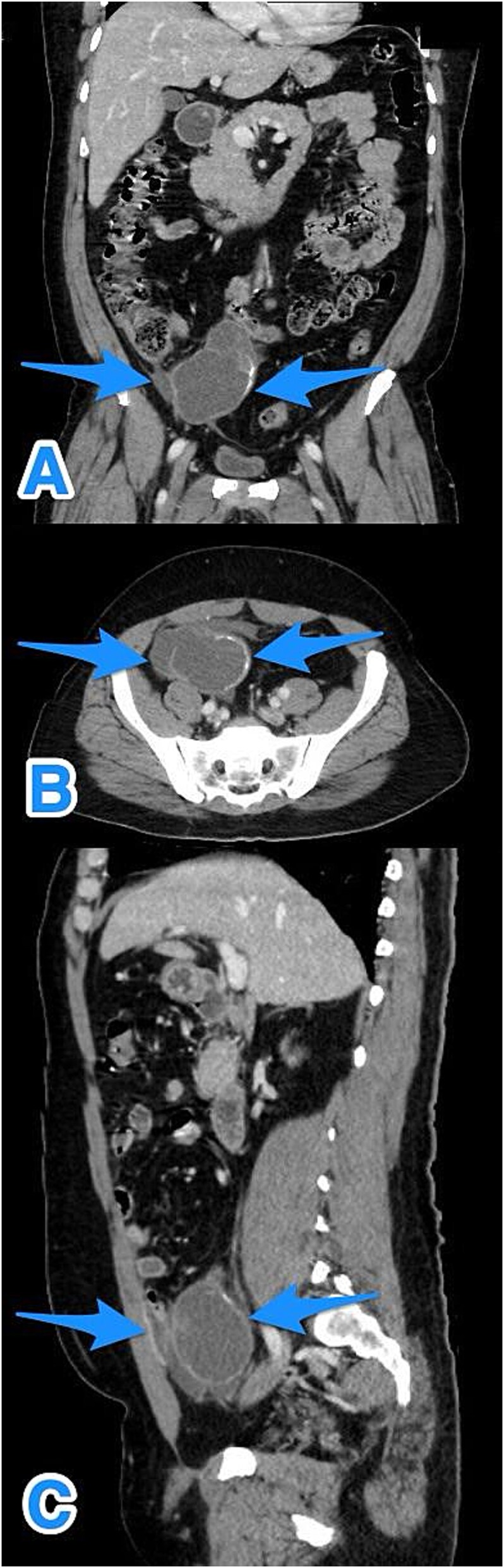
Multiview contrast-enhanced CT images of the perforated appendix with associated free fluid: (A) coronal view, (B) axial view, and (C) sagittal view (arrows).

A contrast-enhanced computed tomography (CT) scan of the abdomen and pelvis demonstrated a fluid-filled, markedly dilated appendix (5.2 cm) with peripheral calcification, particularly at the tip. A focal wall defect was observed on the anterior aspect of the appendix, with associated loculated free fluid, suggestive of rupture (Fig. 1). No significant lymphadenopathy, hepatic, or peritoneal disease was identified. These findings were consistent with a ruptured appendiceal mucocele with localized peritoneal fluid.

**Figure 2:**
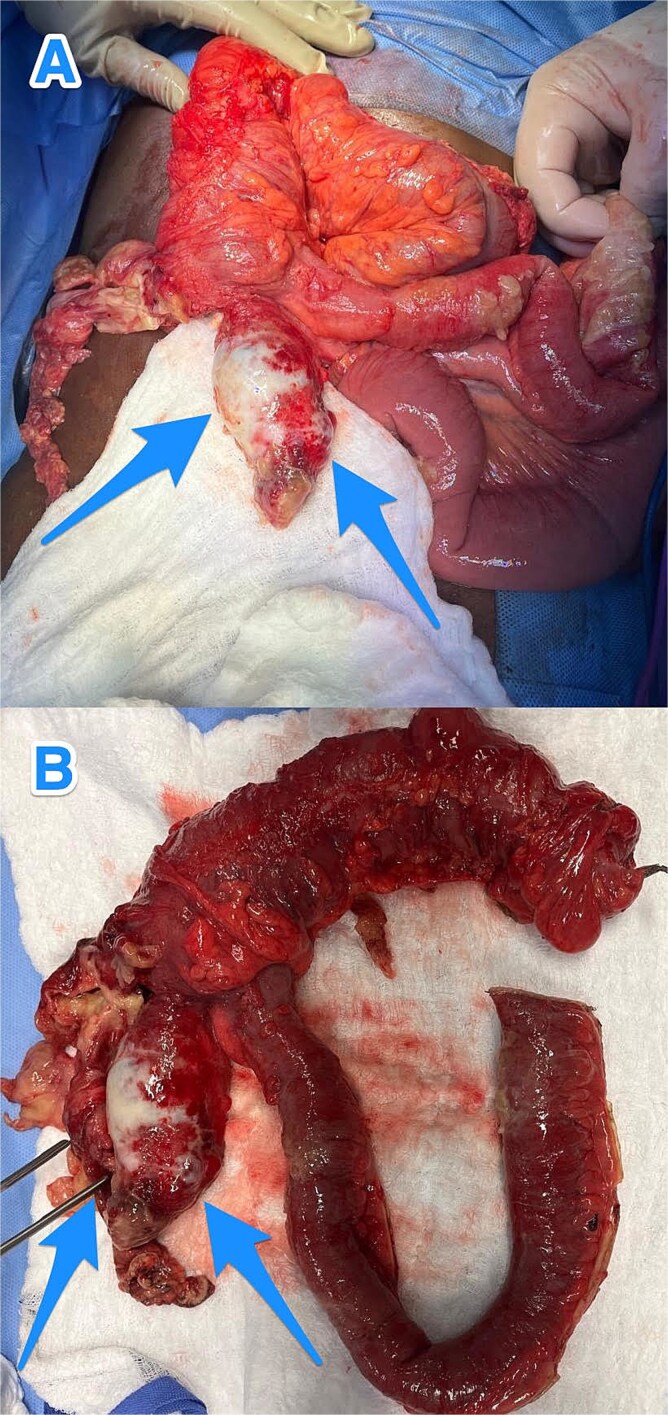
Intraoperative views (A and B) showing an inflamed, dilated, and ruptured appendix with mucin present at the tip (arrows).

Given the localized perforation and the patient’s persistent symptoms, he underwent exploratory laparotomy with right hemicolectomy, peritoneal washout, and closure, as the localized peritoneal disease was cleared intraoperatively (Fig. 2). The patient had an uneventful postoperative course.

**Figure 3:**
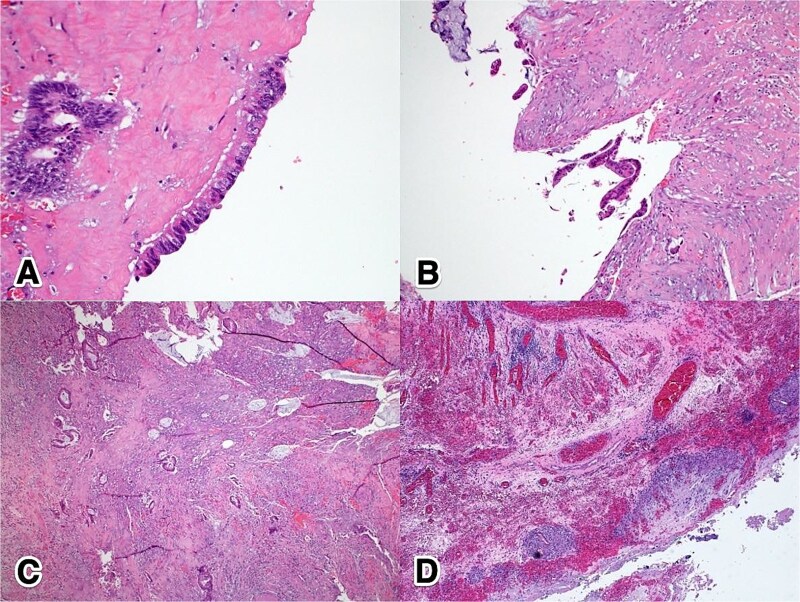
Histopathological features of ANs: (A) 40×, H&E—high-grade dysplastic epithelium at the appendiceal surface; (B) 20×, H&E—high-grade dysplastic epithelium at the appendiceal surface; (C) 4×, H&E—invasive adenocarcinoma penetrating the appendiceal wall; (D) 4×, H&E—acellular mucin with a surrounding granulomatous reaction at the serosal surface.

Histological examination revealed foci of moderately differentiated invasive adenocarcinoma with extracellular mucin production, arising in a background of flat mucinous epithelial proliferation exhibiting high-grade nuclear atypia, consistent with HAMN. Additionally, extensive mucin dissection was observed through the appendiceal wall, extending into the serosa and mesoappendix. The tumor was staged as IIB (pT4aN0M0) (Fig. 3).

Following the final diagnosis of perforated mucinous adenocarcinoma of the appendix with HAMN, the patient was referred to a higher-level center. A CT scan of the chest was negative, and his carcinoembryonic antigen (CEA) level was 1.9. After multidisciplinary team review, the plan was to initiate chemotherapy, as the disease appeared confined to the right lower abdomen and all grossly visible tumor had been resected based on intraoperative findings and follow-up imaging. However, the patient elected to decline chemotherapy and opted for surveillance only (the colorectal cancer surveillance protocol).

## Discussion

Epithelial ANs are rare and often mimic acute appendicitis, typically discovered incidentally during surgery, colonoscopy, or histopathological examination [1]. Symptoms may include vague abdominal pain or signs of obstruction, while a palpable mass is uncommon and suggests advanced disease. The differential diagnosis is broad and includes mucinous neoplasms, Crohn’s disease, and tuberculosis [4].

ANs exhibit diverse histologic subtypes—such as colonic-type adenocarcinoma, mucinous neoplasm, goblet cell carcinoma, and neuroendocrine neoplasm [1]. Rarely, multiple primary tumors coexist as composite or collision tumors [5]. Appendiceal mucinous tumors and adenocarcinomas have prevalences of 0.9% and 0.1%, respectively [6, 7], with dual pathologies found in 4% of cases in a recent Saudi study [8]. No prior cases have reported concurrent HAMN with appendiceal adenocarcinoma. Rare instances include low-grade appendiceal mucinous neoplasm coexisting with a carcinoid tumor [2] or a grade 1 goblet cell adenocarcinoma (pT3) [3].

Abdominal ultrasound is a useful first-line imaging tool [9], while CT is the primary modality for detecting appendiceal mucinous neoplasms, aiding in diagnosis and staging by identifying wall thickening, calcifications, or peritoneal mucin deposits [4, 10]. Colonoscopy can detect appendiceal orifice lesions and rule out synchronous colorectal tumors [4], sometimes revealing the ‘volcano sign’ [11]. CEA helps monitor recurrence, while CA-125 and CA 19-9 may aid diagnosis when the tumor’s origin is unclear, though not routinely recommended [4]. Recent artificial intelligence models now enhance appendiceal cancer prediction using Shapley Additive Explanations (SHAP) and mortality risk estimation [12, 13].

HAMNs are typically non-metastatic; however, when invasive features are present, they are reclassified as mucinous adenocarcinomas [4]. Surgical management of ANs varies by subtype, grade, and stage, ranging from appendectomy to cytoreductive surgery with or without heated intraperitoneal chemotherapy for peritoneal spread [1, 14]. In acute settings, appendectomy or right hemicolectomy is usually performed. In the present case, the patient underwent open right hemicolectomy and was found to have high-risk stage IIB disease. He opted for surveillance instead of adjuvant chemotherapy, which is typically guided by colon cancer protocols given the limited data on ANs. Notably, systemic chemotherapy may benefit patients with metastatic HAMNs. The prognosis of mucinous appendiceal adenocarcinoma is considered intermediate and is primarily influenced by lymph node involvement, distant metastases, and tumor differentiation, rather than perforation, with recent data suggesting similar survival outcomes for mucinous and non-mucinous subtypes [15]. Given the lack of specific surveillance guidelines, further research is warranted to establish standardized follow-up protocols for this tumor type.

## Conclusions

Concurrent ANs, such as adenocarcinoma arising from HAMN, are rare and often mimic acute appendicitis, complicating diagnosis. This first reported case highlights the need for clinical suspicion in atypical presentations, especially when imaging shows mucinous features or appendiceal dilation. Long-term follow-up is crucial, and further research is needed to clarify incidence and improve diagnosis and management.
